# A randomized controlled trial of multi-session online interpretation bias modification training: Short- and long-term effects on anxiety and depression in unselected adolescents

**DOI:** 10.1371/journal.pone.0194274

**Published:** 2018-03-15

**Authors:** Leone de Voogd, Reinout W. Wiers, Peter J. de Jong, Robert J. Zwitser, Elske Salemink

**Affiliations:** 1 Department of Psychology, University of Amsterdam, Amsterdam, The Netherlands; 2 Department of Clinical Psychology, University of Groningen, Groningen, The Netherlands; Brown University, UNITED STATES

## Abstract

**Introduction:**

Negatively biased interpretations play an important role in anxiety and depression, which are highly prevalent in adolescence, and changing such biases might thus reduce or prevent emotional disorders. We investigated the short- and long-term effects of an online interpretation bias modification training in unselected adolescents to explore its potential in preventing anxiety and depression.

**Methods:**

Participants (N = 173) were randomly allocated to eight online sessions of interpretation or placebo training. Interpretation bias was assessed pre- and post-training. Primary outcomes of anxiety and depression, and secondary measures of emotional resilience were assessed pre- and post-training and at three, six, and twelve months follow-up.

**Results:**

Compared to placebo, interpretation training marginally increased positive interpretations. Irrespective of training condition, symptoms of anxiety and depression showed a decline post-training and at follow-up, and indices of resilience showed an increase. Change in interpretation bias, baseline interpretation bias, stressful life events, or number of training sessions completed did not moderate the effects on anxiety or depression.

**Conclusions:**

Results suggest that interpretation training as implemented in this study has no added value in reducing symptoms or enhancing resilience in unselected adolescents.

## Introduction

Cognitive models of anxiety and depression assume that biases in information processing play an important role in the aetiology of these disorders (e.g. [1], [2]). Recently developed cognitive training paradigms directly target such cognitive vulnerabilities and could be employed online as a possible low barrier early intervention or prevention program [3]. Adolescents seem a particularly relevant target group for this type of preventive interventions for two reasons: first, this age-group is the most vulnerable for the development of anxiety and depression [4], and second, it is also in a period of heightened brain plasticity [5]. When a positive information processing style could be acquired at this age, this might protect against the development or worsening of emotional problems. The aim of the current study was to investigate the short- and long-term effects of a specific type of cognitive training: Cognitive Bias Modification for Interpretations (CBM-I), on the following outcomes: interpretation bias, symptoms of anxiety and depression (primary outcomes), and secondary measures of emotional resilience.

There is ample evidence indicating that individuals with anxiety disorders or depression are characterized by a tendency to interpret ambiguous information in a negative way (for a review, see [2]). It has been shown that experimentally increased negative interpretation bias also strengthens people's emotional responding to experimental stressors [6], supporting the relevance of such negative interpretation bias as a causal agent in the development of emotional disorders. These findings fuelled research into potential therapeutic applications of this CBM-I paradigm. In the most-often used CBM-I training paradigm, participants read ambiguous scenarios, which are consistently disambiguated in a positive way by completing a word fragment. Recent meta-analyses of CBM-I studies in adults [7], [8] showed consistent positive effects on interpretation bias, while findings on mood, stress-reactivity, and anxiety or depressive symptoms were more mixed. Mood effects seemed to be larger when CBM-I was used with imagery instructions and with more training sessions [8]. In the context of depression, positive effects were observed with a scenario-based paradigm with more emphasis on imagery (e.g. [9], [10], [11]).

A first meta-analysis on CBM-I in youth [12] revealed a comparable pattern: a significant effect on interpretation bias (with moderate effect size), but no significant overall effects on anxiety and depression. Effects were found to be larger in unselected youth and when training was performed at school. Note that in this meta-analysis, both CBM-I and CBM for attention studies were included. In CBM for attention procedures, participants are trained to focus their attention on positive or neutral information instead of negative or threatening information, in order to reduce a negative attentional bias. Focusing on CBM-I only, a recent re-analysis of six youth studies [13] reported significant mood effects when comparing positive and negative training. Given the large variability in study design (number of sessions, sample, type of training, and assessment tasks) and the small number of included studies and participants, the results from both meta-analyses are difficult to interpret. Important steps forward are performing larger studies with more power to detect effects and explore for whom training works best. Earlier research suggested that training might be especially effective in those adolescents with a more negative interpretation bias [14], [15], but some studies employing multiple sessions of CBM-I training in healthy adolescents also showed changes in stress responses [16], [17]. The current study focused on unselected adolescents, varying from no symptoms to a clinical level of anxiety or depressive symptoms. This provides the possibility to examine which adolescents profit most, and to test the effects on symptomatology as well as on resilience (e.g. stress-reactivity, self-esteem), thus exploring the potential of CBM-I as a universal or targeted prevention program. Based on meta-analytic findings, training was optimized by the use of multiple sessions and more emphasis on imagery.

CBM-I training has a clear hypothesized mechanism of change; potential emotional effects should be mediated by change in interpretations. Only a few studies have directly tested the hypothesized mediational path and found that change in interpretations indeed (partly) mediated change in depressive symptoms [11], trait anxiety [18], and social anxiety [19]. Note that it is also quite likely that for the changed interpretation bias to affect emotional functioning, time is needed to apply the new processing style in daily life. That is, to interpret (stressful) life experiences in a more positive way and to change behaviour correspondingly [20]. Therefore, to assess processes of change and to fully appreciate the potential of CBM-I, multiple assessments over a longer period are crucial.

Until now, most research has focused on short-term effects (pre-post-design), with only a handful of studies including follow-up assessments after several weeks or months. In adults, marginally significant effects on social anxiety were observed four weeks after CBM-I training [21] and significant effects at seven weeks follow-up [22]. However, Salemink, Kindt, Rienties, and van den Hout [23] found no effects at three months follow-up, but also no short-term effects were observed in that study. The only RCT that investigated CBM-I as an early preventive intervention in adolescents focussed on youngsters with heightened levels of social and/or test anxiety and used a 10-week internet-based multi method approach including both CBM-I and an attentional bias training. Although the multi method CBM intervention showed a positive effect on interpretation bias that was still evident at two-year follow up, this study failed to find convincing evidence for the efficacy of the combined training to reduce symptoms of social and test anxiety [24][25].

In the current study, adolescents were randomized over one of two training groups: a CBM-I training or a placebo-control training, consisting of eight online sessions, completed over four weeks. Interpretation bias was assessed during training, and pre- and post-training (recognition task). Emotional measures were administered both pre- and post-training, and at three, six, and 12 months follow-up. Our first, and primary hypothesis was that CBM-I would reduce symptoms of anxiety and depression compared to a placebo training, both at the short- and long-term. Second, compared to placebo, we expected a stronger reduction in negative interpretation bias in the CBM-I group. Our third hypothesis was that symptom change would be larger for participants who showed a larger change in interpretation bias. Fourthly, we examined other factors that moderated training effectiveness. More specifically, we tested whether stronger training effects would be observed in adolescents with a more negative baseline interpretation bias, or in adolescents who experienced a relatively large amount of real life stress. Also, we investigated whether training effects would be stronger when completing a larger number of training sessions (cf. [26]). Finally, to further explore the preventive potential of CBM-I in increasing emotional resilience, we assessed immediate effects on stress-reactivity, as well as short and long-term effects on secondary emotional measures of self-esteem, perseverative negative thinking, test anxiety, and social-emotional, and behavioral problems.

## Methods

### Design and ethics

The current study was approved by the ethics committee of the psychology department of the University of Amsterdam and carried out in accordance with the provisions of the Declaration of Helsinki. It was part of a larger study, which was registered in the Dutch trial register with number NTR3950 (http://www.trialregister.nl/trialreg/admin/rctview.asp?TC=3950), and also included two types of attentional bias training, an emotional working memory training and three corresponding placebo groups. The focus of the current manuscript is on the CBM-I and CBM-I placebo training and results of the other paradigms are reported in separate papers [27], [28]. Manipulations and measures not used in this specific study are described in S1 Appendix. The trial was registered after the start of participant recruitment but before the end of data collection, as trial registration of experimental preventive trials was not yet standard policy at our department.

Randomization was performed for the larger study, stratified by school, gender and age group (under/above 15 years), and followed a 4:4:4:4:1:1:1:1 ratio, with four experimental and four placebo conditions respectively. Fewer participants were randomized to the placebo conditions (a total of 20%), to increase the appeal of the project to schools, and because we originally planned to combine them into one control condition (resulting in five conditions). The computerized randomization procedure was written by a programmer not involved in the study, and both participants and test assistants were blind to allocation.

An a-priori power analysis was performed for the larger study in G*Power 3.1 [29]. The required sample size to detect a within-between interaction in a repeated measures ANOVA was computed with the following parameters: a small effect size of *f* = .10 (based on [30], [31], [32]), a power of 90%, an alpha of .05, five groups, five measurements, a correlation of 0.5 between measurements, and a nonsphericity correction of 0.375. The power analysis revealed that 470 participants were needed in order to detect a Condition (five groups: CBM-I, combined control condition, and the three other experimental conditions, see above) x Time interaction effect in predicting anxiety or depression scores (our primary outcome measures). Anticipating drop-out, we aimed for 600 participants. Since training compliance was relatively low in the first ten schools (five out of eight sessions completed on average), four more schools were invited to increase the expected number of completers (resulting in a total of 14 schools). Recruitment stopped after including participants from those four schools.

Note that the a-priori power analysis was performed for five groups, as we initially planned to analyze all placebo groups together as one combined control group and compare it with all experimental groups. As we decided to focus in this paper on interpretive bias only and thus perform analyses for the CBM-I and CBM-I Placebo group specifically, with stringent corrections for multiple comparisons, this has reduced our power. A sensitivity analysis with 2 groups, a sample size of 78 (based on our smallest group), 5 measurements, a correlation of 0.5, a nonsphericity correction of 0.25 (unstructured covariances), an alpha of .0045 (after Bonferroni Holm correction), and a power of .90, revealed that we were able to detect a Condition x Time interaction with a medium effect size of f = .30.

### Participants

**Fig 1:**
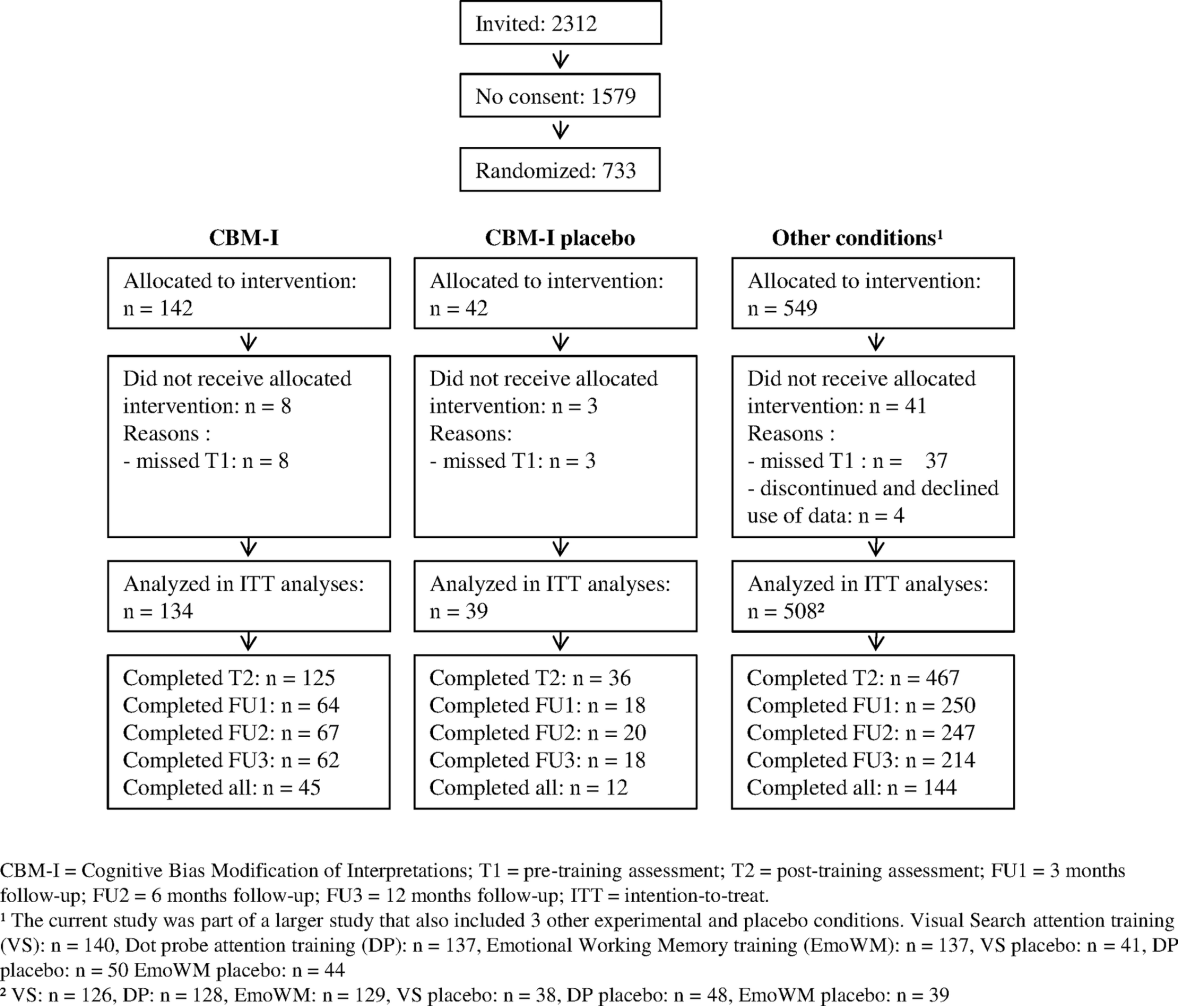
Flow chart of the larger study.

In total, 2312 adolescents from 14 regular high schools in the Netherlands were invited for the study between January and September 2013. The last follow-up assessments were completed in November 2014. Inclusion criteria were: Scholars in the 1^st^ to 6^th^ grade (aged 11–19) of a regular high school (all levels except special education), and parental consent. A total of 733 participants and their parents provided written informed consent (see Fig 1 for flow diagram of the larger study) and were randomized. Four participants dropped out and were excluded since they requested removal of their data and 48 participants were excluded because they missed the first assessment, resulting in a total of 681 participants for the larger study. For the CBM-I and CBM-I Placebo (from now on referred to as ‘Placebo’) group, 173 participants (134 and 39 respectively) remained for intention-to-treat analyses (60.7% female, mean age 14.35, *SD* = 1.11). Background variables of these groups can be found in Table 1.

**Table 1:** Demographic characteristics per training condition.

	CBM-I (n = 134)	Placebo (n = 39)
Age, mean (*SD*)	14.31 (1.10)	14.49 (1.16)
Female, n (%)	82 (61.19)	23 (58.97)
School level, n (%)		
- *Lower*	32 (23.88)	11 (28.21)
- *Middle*	28 (20.90)	6 (15.38)
- *Higher*	74 (55.22)	22 (56.41)
Sessions, mean (*SD*)	5.54 (2.33)	5.38 (2.35)
High life events group, n (%)	40 (29.90)	16 (43.20)

### Interpretation training (CBM-I)

The CBM-I paradigm from Mathews and Mackintosh [6] was used to manipulate interpretation bias. Participants read 3-line ambiguous scenarios, with a missing word in the last sentence, presented as a word-fragment. Participants had to indicate the first missing letter with the corresponding key, after pressing the spacebar as quickly as they recognized the word. Completing the word-fragment disambiguated the scenario in a positive way in experimental trials. The correct word was displayed after a correct response and the interpretation was reinforced by a “yes” or “no” comprehension question about the scenario, followed by feedback. An example scenario might be: “You are playing a solo as part of a concert. As you are playing you know you are making some mistakes. At the end you think back to the bits that you played well and feel pl—sed (pleased)”. “Do you feel happy when you think about the bits you played well? (Yes)”. Each training session consisted of three blocks of 14 trials, with 10 training scenarios and two positive and two negative probe scenarios (disambiguated in a positive or negative way respectively). The order of scenarios was randomized beforehand, with the same order applied to all participants. Probe scenarios were used to assess interpretation bias during training and also obscured the goal of training [6]. A relative reduction in reaction times to positive probes compared to negative probes would indicate a decrease in negative interpretation bias. Participants were asked to imagine the scenarios as happening to themselves and as vividly as possible, as the use of mental imagery has been found to increase training effects [25]. After each 4^th^ trial, participants rated to what extent they were able to imagine the outcome of the scenario on a 4-point scale. In the Placebo condition, the 10 training scenarios were in the same context and started with the same sentence, but ended in a neutral way. Comprehension questions focused on factual information. The same probe scenarios as in the experimental condition were used.

In total, 576 unique ambiguous scenarios (with 288 positive, 240 neutral and 48 negative resolutions) were created, based on previous studies in the context of anxiety and depression [9], [15], [23], [33], [34]. Scenarios previously used with adults were adapted for adolescents and English scenarios were translated and adapted to Dutch culture where appropriate. For scenarios including gender-specific words (e.g., your boyfriend/girlfriend) male and female versions were created. New scenarios were also developed for situations specific to panic, generalized anxiety, and depressed mood, as most original scenarios focused on social situations.

A progress bar indicated how many trials were left in each block. Between blocks, short breaks were provided with feedback, consisting of the number of points earned based on performance (one point for each correct answer, to word fragments and comprehension questions). At the end of each session, points of this and previous session(s) were presented in a graph. We expected this feedback to improve motivation and engagement (cf. [35]).

### Primary outcome measures

Anxiety symptoms were assessed with the Screen for Child Anxiety Related Emotional Disorders (SCARED, [36]), a 41-item (rated 0–2) self-report questionnaire assessing social phobia, separation anxiety, generalized anxiety, panic/somatic symptoms and school phobia.

Depressive symptoms were assessed with the Children’s Depression Inventory (CDI, [37]), a 27-item self-report questionnaire with items consisting of three statements indicating varying levels of depressive symptomatology (0–2).

### Secondary cognitive outcome measures

Interpretation bias was assessed with the Recognition Task (REC-T, [6]), where participants read ambiguous scenarios, completed word-fragments and answered comprehension questions as in the CBM-I training. However, here the scenario remained ambiguous also after completing the word fragments. After presentation of eight scenarios, titles of these scenarios were presented again in random order, once with a negative interpretation and once with a positive interpretation (randomized). Participants rated the extent to which the interpretation corresponded to the scenarios on a 4-point scale. An interpretation bias index was computed by subtracting ratings for positive interpretations from ratings for negative interpretations; a higher score thus indicated a negative interpretation bias. Two stimuli sets were created to use pre- and post-training, and they were counterbalanced across participants. The REC-T has been used repeatedly to assess effects of CBM-I in adolescents (e.g., [14], [15], [16], [17], [24]), and has been shown to differentiate between high and low neuroticism in adults, while scores are not affected by mood state [38].

### Secondary emotional outcome measures

Self-esteem was assessed with the Rosenberg Self-Esteem Scale (RSES, [39]), a 10-item (rated 1–4) self-report questionnaire.

Test anxiety was assessed with a Dutch self-report questionnaire, the “performance motivation test for children” (Prestatie Motivatie Test voor Kinderen, PMT-K, [40]). Only the 14-item (rated 0–1) subscale assessing negative test anxiety was used.

The Perseverative Thinking Questionnaire (PTQ, [41]) was used to assess worry and rumination. The PTQ is a 15-item (rated 1–5) self-report questionnaire assessing key features of repetitive negative thinking (repetitive, intrusive and difficult to disengage from) and the unproductiveness of and mental capacity captured by this thinking.

The Strengths and Difficulties Questionnaire (SDQ, [42]) is a 25-item (rated 0–2) self- and parent-report questionnaire assessing emotional problems, conduct problems, hyperactivity-inattention and peer problems as well as pro-social behaviour. The total difficulties score, computed based on all problem subscales, was used in this study.

Stress reactivity was assessed by using Cyberball [43], [44] as a social stressor. In this task, participants are led to believe that they play an online ball-tossing game, which is programmed such that after two own tosses, the participant is excluded from the game. To assess changes in mood in response to the stress-task, participants had to indicate how anxious, nervous, sad, happy, confident, and enthusiastic they felt on a scale from 0–100 (not at all–very much) before and after the task. Ratings were combined into a positive and negative mood scale respectively.

Internal consistency for all emotional outcome measures was adequate to excellent in the larger study sample (SCARED *α* = .92, CDI *α* = .86, RSES *α* = .86, PTQ *α* = .95, PMT-K *α* = .81, SDQ *α* = .71, SDQ-parent *α* = .71, positive mood *α* = .72, negative mood *α* = .65).

### Stressful life events

Stressful life events were assessed with the Dutch “TRAILS events scale” (‘TRAILS Gebeurtenissen vragenlijst’, [45]), a self-report questionnaire assessing the occurrence and impact of 25 stressful events (e.g., parental divorce, severe illness/death of a family member, victimization). Participants had to indicate whether the event occurred either during the past three months, during the last two years or never/longer ago and how stressful (rated 0–3) the experienced event was. A stressful life events index was calculated by adding the impact scores for all life events that had been experienced in the previous period. Next, based on [45] we dichotomized this index into “high stress” (scores > 6) and “low or average stress” for each time point. Finally, since we were interested in the long-term interaction between stress and training, groups were created separating those who were in the “high stress” group at least at one time point and those who never were.

### Procedure

Adolescents of invited classes received oral instructions about the content and aim of the study, explained as “investigating a training to make adolescents more resilient to stress and negative emotions, by learning to worry less and have a more positive view on your environment”. Both adolescents and parents received information letters and had to provide written informed consent. The first assessment (T1), the first training and the post-training assessment (T2) were completed under supervision and took place during regular school hours in a computer classroom. Assessments started with the REC-T and some other computer tasks, followed by the online questionnaires, and participants were automatically directed to the next task. Apart from interpretation bias, also attentional bias and working memory were assessed, and questionnaires on attentional control, alcohol-related problems and high sensitive personality were administered, but not used for the current study. See S1 Appendix for a description of these materials. Both assessments took about 80 minutes. One to seven days after T1, the first training session was performed at school. For the remaining training sessions, participants received a reminder by e-mail and text message twice a week. Each session took approximately 15 minutes and had to be completed within two days. Reminders were sent after missing two sessions, offering technical assistance where needed. T2 was almost identical to T1, except for the inclusion of the Cyberball stress-task, and took place 1–7 days after the last training sessions. At the end of this session, participants were fully debriefed on Cyberball and compensated by vouchers and participation in a lottery, based on the number of sessions completed (<six sessions: one lottery ticket, six or seven sessions: five euros and two lottery tickets, eight sessions: 10 euros & three lottery tickets). Three (FU1), six (FU2) and 12 (FU3) months after T2, participants received a text message and an e-mail with a link to complete the follow-up assessments, consisting of the same questionnaires as T1. Reminders were sent after one week, and test-assistants made phone calls to non-responders after two weeks.

### Data analyses

To examine whether the CBM-I and Placebo group differed on demographic characteristics or baseline scores on outcome measures, chi-square tests and independent t-tests were performed.

To assess potential treatment effects, mixed regression analysis was performed. This method takes into account repeated assessments and uses all available data without discarding participants with missing data at specific time points. For all outcome measures, a mixed model with Participant as the grouping variable and Time as a repeated measure variable was tested. With regard to the covariance between time points, we verified (based on AIC and BIC criteria) whether these were structured according to compound symmetry, or first order autoregressive, or whether these were unstructured. The latter was the case for all analyses. School could have been added as another grouping variable, but was not included, as preliminary analyses indicated that this did not improve model fit, and that school explained less than 0.6% of the variance in our primary outcome measures.

To test our first and second hypothesis, for both anxiety and depressive symptoms, and interpretation bias (REC-T and RTs to probe scenarios) respectively, a model including the factors Time and Condition (CBM-I or Placebo), and their interaction, was created. The factor Time had two levels for short-term outcome measures (T1 and T2), five levels for long-term measures (T1, T2, FU1, FU2, and FU3) and eight levels for probe RTs (one for each training session). The best model was selected in a backward elimination procedure, in which parameters were excluded from the model based on AIC and BIC criteria and significance level of the parameters.

To test our third hypothesis, i.e. that symptom change would be larger for participants who showed a larger change in interpretation bias, a model was created including the factors Time and Condition, the covariate bias change and all possible interaction terms. Note that while conceptually we hypothesized change in interpretation bias to be a mediator of emotional effects, in mixed regression this was implemented as a moderating factor, as this analysis method is more suitable here, and mediation also implies that emotional effects will be observed specifically in those participants who display a change in bias. To test our fourth hypothesis, we assessed the moderating role of baseline interpretation bias, stressful life events, or number of completed training sessions with separate models using the same approach. The effects of interest in these analyses were the three-way interactions between Time, Condition, and Moderator.

To explore training effects on stress reactivity, and the other secondary outcome measures (RSES, PTQ, PMT-K, SDQ, SDQ-P, mood scales), the same procedure as for the primary outcome measures was used, starting with a model including Time, Condition and their interaction.

To control for Type I errors related to the number of outcome measures, Bonferroni-Holm correction was applied for the full set of 11 outcome measures. Effects with *p* <05 that did not survive this correction were defined as marginal.

## Results

### Preliminary analyses

The CBM-I and Placebo group did not differ on demographic characteristics nor outcome measures at baseline, all *p’s* > .320. On average, participants completed 5.51 sessions (*SD* = 2.32), and 41 adolescents (23.7%) completed all eight training sessions. Girls completed significantly more training sessions than boys, *t* (131.71) = -3.34, *p* = .001. Missing data ranged between 7.5% at T2 (17.9% for parent-report), and 54.3% at FU3 (41.6% for parent-report). The number of completed assessments was not related to training condition, nor baseline scores on any of the outcome measures, all *p’s* > .136. However, girls completed more assessment sessions than boys, *χ*^*2*^ (4) = 9.83 *p* = .043.

The REC-T scores at baseline were significantly smaller than zero, *t* (171) = -7.34, *p* <.001, indicating that at baseline participants generally showed a positive interpretation bias. Interpretation bias was not correlated with anxiety or depressive symptoms, *r* = .11, *p* = .147, and *r* = .13, *p* = .093, respectively. Table 2 shows descriptive statistics for both training groups for all outcome measures. Statistics of the original and final models for all hypotheses can be found in Table 3, and Table 4 shows the relevant parameter estimates.

**Table 2:** Outcome measures per training condition.

Condition	Outcome measure^a^	T1^b^ pre-training assessment		T2 post-training assessment		FU1 3 months follow-up		FU2 6 months Follow-up		FU3 12 months follow-up	
		*M*	*SD*	*M*	*SD*	*M*	*SD*	*M*	*SD*	*M*	*SD*
CBM-I (n = 134)	REC-T	-0.32	0.58	-0.93	0.77	-	-	-	-	-	-
	SCARED	20.31	12.13	17.57	12.73	18.15	12.01	16.73	12.37	16.30	12.35
	CDI	8.68	5.54	7.70	6.52	7.34	5.76	6.68	6.42	6.10	6.13
	Positive mood	200.52	62.04	198.57	71.46	-	-	-	-	-	-
	Negative mood	41.00	48.36	38.42	45.90	-	-	-	-	-	-
	RSES	30.14	4.88	31.19	5.27	31.21	5.05	32.04	5.14	31.84	5.27
	PTQ	35.18	12.27	34.14	12.61	32.78	11.18	33.55	14.02	32.16	13.50
	PMT-K	7.66	3.59	7.05	3.66	7.20	3.35	6.73	3.79	5.98	3.62
	SDQ	10.69	5.12	10.04	5.35	9.35	5.51	9.21	5.74	8.25	5.13
	SDQ-parent	6.44	5.06	6.34	5.32	5.61	4.6	5.05	4.33	5.09	4.18
Placebo (n = 39)	REC-T	-0.34	0.56	-0.60	0.60	-	-	-	-	-	-
	SCARED	18.15	10.85	16.00	10.04	17.67	9.36	15.60	8.12	14.39	7.55
	CDI	8.87	6.42	8.14	5.63	5.72	4.21	8.10	6.36	7.61	6.60
	Positive mood	201.53	59.11	201.29	65.03	-	-	-	-	-	-
	Negative mood	47.11	44.19	37.63	44.78	-	-	-	-	-	-
	RSES	29.62	4.85	29.89	5.69	31.16	5.21	29.55	4.91	30.37	5.62
	PTQ	36.26	12.91	32.33	12.12	32.44	9.96	32.35	13.14	29.28	12.21
	PMT-K	7.97	3.32	7.28	3.28	7.94	3.84	8.10	3.45	7.11	4.34
	SDQ	9.82	5.43	9.11	4.32	8.37	3.34	9.05	5.92	7.39	4.07
	SDQ-parent	7.32	5.07	6.42	4.76	6.37	4.38	6.46	5.39	7.16	5.34

^a^ REC-T = Recognition Task; SCARED = Screen for Child Anxiety Related Emotional Disorders; CDI = Children’s Depression Inventory; RSES = Rosenberg Self-Esteem Scale; PTQ = Perseverative Thinking Questionnaire; PMT-K = Performance Motivation Test for children; SDQ(-P) = Strengths and Difficulties Questionnaire (Parent)

^b^ Note that for positive and negative mood, T1 and T2 refer to pre- and post-stressor mood respectively, both assessed at the post-training assessment session.

**Table 3:** Statistics of the original and final models for all hypotheses.

Outcome measure ^a^	Model ^b^	Model fit		Time		Condition		Condition * Time		Condition * Time * Moderator^c^	
		*AIC*	*BIC*	*F*	*df*	*F*	*df*	*F*	*df*	*F*	*df*
REC-T	**Condition** * **Time**	662.37	689.00	27.95***	1, 165.15	2.69	1, 166.90	4.40*	1, 165.15	-	-
Bias index probes	Condition * Time	13009.71	13258.79	15.68***	7, 111.48	16.23***	1, 143.61	1.23	7, 111.48	-	-
	**Condition + Time**	13003.89	13219.44	22.53***	7, 108.59	14.13***	1, 115.99	-	-	-	-
Negative probes	Condition * Time	13604.12	13855.77	21.10***	7, 106.79	0.94	1, 159.57	1.01	7, 106.79	-	-
	**Time**	13596.64	13809.58	31.98***	7, 106.64	-	-	-	-	-	-
Positive probes	**Condition** * **Time**	13661.39	13913.10	49.51***	7, 109.89	7.29	1, 164.33	2.99**	7, 109.89	-	-
SCARED	Condition * Time	4169.85	4278.97	5.92***	4, 112.69	0.31	1, 165.17	0.30	4, 112.69	-	-
	**Time**	4161.45	4248.75	9.87***	4, 111.99	-	-	-	-	-	-
	Condition * Time * Bias change	3994.09	4145.51	4.86**	4, 108.81	0.07	1, 152.12	0.61	4, 108.81	0.41	4, 111.28
	Condition * Time * Bias	4140.06	4292.52	6.20***	4, 111.63	0.30	1, 165.79	0.31	4, 111.63	0.14	4, 116.08
	Condition * Time * Life events	4163.99	4316.75	5.17**	4, 120.80	0.71	1, 172.59	0.29	4, 120.80	0.29	4, 120.80
	Condition * Time * Sessions	4175.90	4328.67	5.80***	4, 148.77	0.63	1, 192.33	0.31	4, 148.77	1.23	4, 147.64
CDI	Condition * Time	3339.44	3448.47	1.65	4, 91.67	0.28	1, 160.35	0.98	4, 91.67	-	-
	**Time**	3333.20	3420.43	2.57*	4, 91.52	-	-	-	-	-	-
	Condition * Time * Bias change	3195.44	3346.73	1.11	4, 86.70	0.17	1, 146.41	0.91	4, 86.70	0.70	4, 88.82
	Condition * Time * Bias	3316.46	3468.80	1.78	4, 92.25	0.29	1, 157.75	0.87	4, 92.25	0.31	4, 97.63
	Condition * Time * Life events	3319.01	3471.66	2.10	4, 102.16	0.13	1, 164.63	1.28	4, 102.16	1.05	4, 102.16
	Condition * Time * Sessions	3347.13	3499.78	1.87	4, 137.44	0.09	1, 174.91	2.40^†^	4, 137.44	1.38	4, 139.42
Positive mood	Condition * Time	3357.13	3383.42	0.36	1, 156.03	0.01	1, 159.98	0.01	1, 156.03	-	-
** **	**Time**	3353.14	3371.92	0.55	1, 156.03	-	-	-	-	-	-
Negative mood	Condition * Time	3137.53	3163.82	5.08*	1, 157.41	0.13	1, 160.34	1.58	1, 157.41	-	-
	**Time**	3135.17	3153.95	3.46^†^	1, 157.34	-	-	-	-	-	-
RSES	Condition * Time	3305.06	3414.48	1.91	4, 102.41	1.44	1, 158.48	0.77	4, 102.41	-	-
	**Time**	3398.80	3386.33	3.72**	4, 105.65	-	-	-	-	-	-
PTQ	Condition * Time	4232.85	4341.92	6.36***	4, 95.27	0.03	1, 163.50	0.94	4, 95.27	-	-
	**Time**	4226.58	4313.84	6.17***	4, 94.86	-	-	-	-	-	-
PMTK	Condition * Time	2773.03	2882.11	5.69***	4, 100.40	0.91	1, 159.23	0.45	4, 100.40	-	-
	**Time**	2765.36	2852.62	9.04***	4, 100.54	-	-	-	-	-	-
SDQ	Condition * Time	3252.50	3361.71	4.37**	4, 101.22	0.70	1, 165.87	0.17	4, 101.22	-	-
	**Time**	3243.96	3331.33	5.86***	4, 100.91	-	-	-	-	-	-
SDQ-P	Condition * Time	3148.66	3258.91	2.28^†^	4, 115.53	1.40	1, 160.67	0.19	4, 115.53	-	-
	**Time**	3140.77	3228.97	3.18*	4, 115.10	-	-	-	-	-	-

^†^ p < .10

* *p* ** *p* *** *p* Note that most p-values between *p* ^a^ REC-T = Recognition Task; SCARED = Screen for Child Anxiety Related Emotional Disorders; CDI = Children’s Depression Inventory; RSES = Rosenberg Self-Esteem Scale; PTQ = Perseverative Thinking Questionnaire; PMT-K = Performance Motivation Test for children; SDQ = Strengths and Difficulties Questionnaire

^b^ Condition = CBM-I versus Placebo; Time = two levels for REC-T, eight levels for probes, and five levels for all other outcome measures. Bold print = final model, based on AIC and BIC and significance of parameters. Lower AIC and BIC values represent a better model fit. Note that moderation models were tested after testing general training effects on primary outcome measures (SCARED and CDI).

^c^ Moderator refers to the specific potential moderator included in the model (Bias change, Bias, Life events, or Sessions)

**Table 4:** Parameters estimates of significant effects.

*Training effects*		CBM-I ^a^		T2		FU1		FU2		FU2		T2 CBM-I	
		*B*	*SE*	*B*	*SE*	*B*	*SE*	*B*	*SE*	*B*	*SE*	*B*	*SE*
REC-T ^b^	Condition * Time	-	-	-0.26^†^	0.15	-	-	-	-	-	-	-0.35*	0.17
Bias index probes	Condition + Time ^c^	-102.19***	27.19	-	-	-	-	-	-	-	-	-	-
SCARED	Time	-	-	-2.33***	0.54	-2.41*	0.95	-4.22***	0.95	-4.98***	0.97	-	-
CDI	Time	-	-	-0.63*	0.29	-1.19*	0.46	-1.37**	0.50	-1.35*	0.54	-	-
RSES	Time	-	-	0.82**	0.30	1.06*	0.47	1.33**	0.42	1.63**	0.47	-	-
PTQ	Time	-	-	-1.51*	0.59	-2.40*	1.01	-3.42**	1.10	-4.64***	1.02	-	-
PMTK	Time	-	-	-0.61**	0.17	-0.61*	0.30	-1.08**	0.32	-1.75***	0.31	-	-
SDQ	Time	-	-	-0.45^†^	0.26	-1.00*	0.42	-1.12*	0.43	-1.90***	0.40	-	-
SDQ-P	Time	-	-	0.20	0.23	-0.88**	0.28	-1.08**	0.34	-0.83*	0.34	-	-

^†^ p .10

* p < .05

** p < .01

*** p < .001

Note that most p-values between *p* ^a^ Reference categories for parameters estimates were the placebo condition and the pre-training assessment (T1). T2 = post-training assessment; FU1 = 3 months follow-up; FU2 = 6 months follow-up; FU3 = 12 months follow-up.

^b^ REC-T = Recognition Task; SCARED = Screen for Child Anxiety Related Emotional Disorders; CDI = Children’s Depression Inventory; RSES = Rosenberg Self-Esteem Scale; PTQ = Perseverative Thinking Questionnaire; PMT-K = Performance Motivation Test for children; SDQ(-P) = Strengths and Difficulties Questionnaire (Parent)

^c^ Time effects are not included in this Table, since this model included the eight training sessions as time points. Bias index was significantly reduced at all sessions compared to the first session (all *p’s* < .001, parameter estimates between *B* = -489.60, *SE* = 55.51, and *B* = -275.29, *SE* = 57.90), except for the fourth session, *p* = .224.

### Primary outcome measures

Our first and primary hypothesis, that CBM-I would result in reduced anxiety and depressive symptoms compared to Placebo, was not confirmed, as no significant Condition x Time interactions were observed for SCARED and CDI scores. For anxiety a significant main effect of Time was found, *p* < .001, and for depressive symptoms a marginal Time effect, *p* = .043, both indicating a general decrease in symptoms from T1 to T2, that remained significant or marginally significant at all follow-up assessments.

### Secondary cognitive outcome measures

Our second hypothesis, that CBM-I would result in a reduction of negative interpretation bias compared to placebo, was confirmed by a marginal Condition x Time interaction for interpretation bias assessed with the REC-T, *p* = .037. That is, a larger reduction in negative interpretation bias (i.e. a relative increase in positive interpretations) was found in the CBM-I group compared to Placebo.

**Fig 2:**
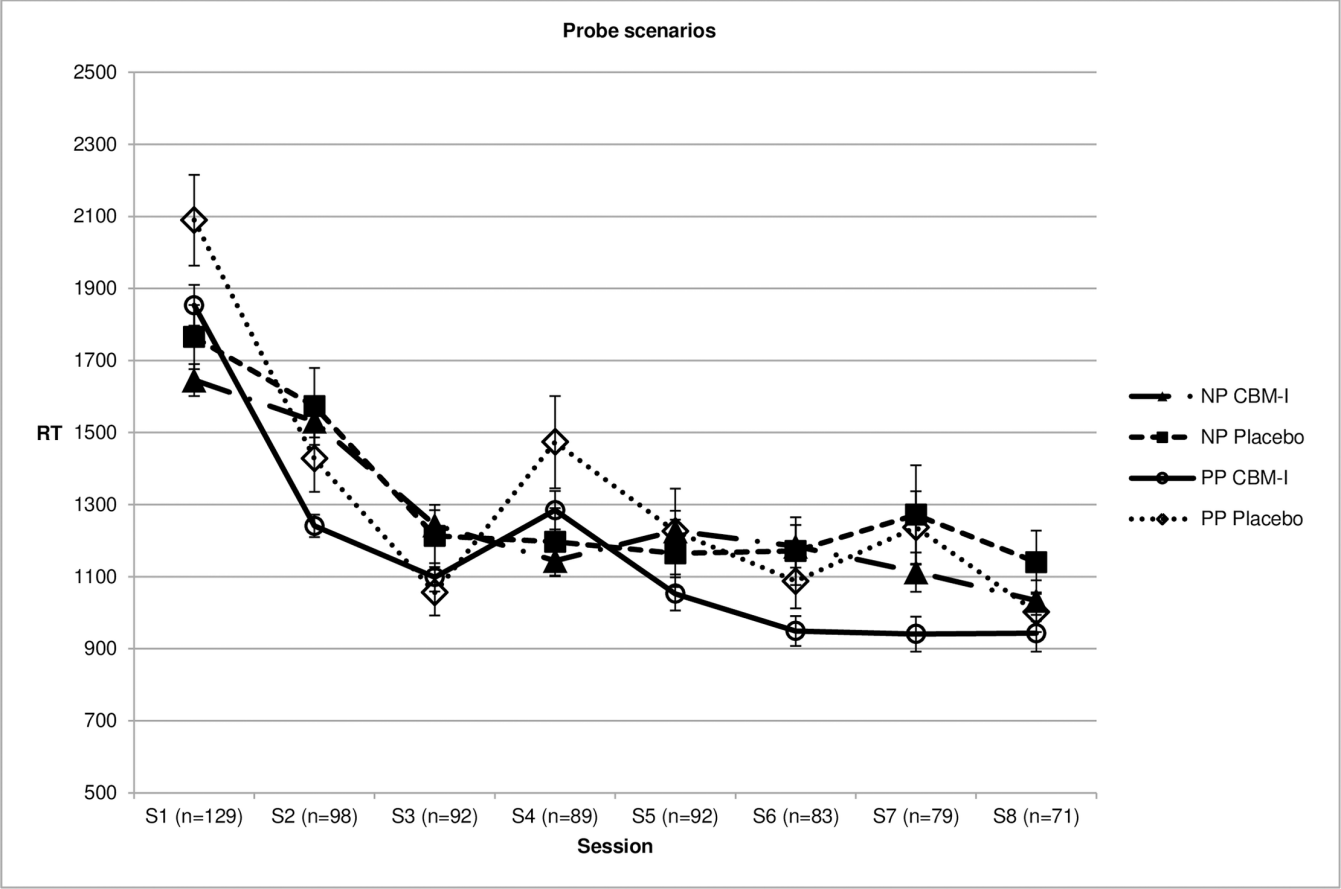
Mean reactions times (RT) to negative probe scenarios (NP) and positive probe scenarios (PP) during training sessions for the two training groups.

For interpretation bias assessed with RTs to probe scenarios (bias index = RTs to positive probes—RTs to negative probes), the expected Condition x Time interaction was not significant, but significant main effects of Condition and Time were found, both *p’s* <.001, indicating a general increase in positive interpretation bias and a more positive bias in the CBM-I group compared to placebo. Testing separate models for negative and positive probes revealed only a significant effect of Time for negative probes, *p* <.001, indicating reduced RTs over time in both groups. For positive probes, the expected Condition x Time interaction was observed, *p* = .007, indicating a significantly larger reduction in RTs to positive probes in the CBM-I group compared to the Placebo group. This indicates that participants who were following the CBM-I training became quicker in responding to positive probes, suggesting quickening of positive interpretations. Fig 2 shows the development of RTs throughout training sessions.

### Moderation of training effects

Our third hypothesis, that training effects on anxiety and depressive symptoms would be larger for participants who showed a larger reduction in negative interpretation bias, was not confirmed, as no three-way interactions between Condition, Time and change in interpretation bias were observed.

Our fourth hypothesis, that training effects on anxiety and depressive symptoms would be moderated by baseline interpretation bias, stressful life events, or number of training sessions completed, was also not supported, as no three-way interactions between Condition and Time, and these potential moderators were observed.

### Secondary emotional outcome measures

Finally we explored training effects on secondary emotional outcomes, that is, stress-reactivity immediately post-training, and self-esteem, perseverative negative thinking, test-anxiety, and social-emotional and behavioural problems post-training and at follow-up. Contrary to our expectations, no changes in mood were observed in response to the stress task, nor was mood affected by training condition. For all other secondary outcome measures, significant Time effects were found, indicating reductions in symptoms, with some variability in the specific comparisons between time points that were significant. No differential training effects were observed between the CBM-I and Placebo group for these measures.

## Discussion

The aim of the current study was to investigate the short- and long-term effects of multiple sessions of online CBM-I on interpretation bias, anxiety, depression, and emotional resilience in unselected adolescents. Our primary hypothesis was that CBM-I would result in reduced symptoms of anxiety and depression compared to placebo. Furthermore, we hypothesized that CBM-I would reduce negative interpretation bias (i.e. enhance positive interpretation bias), and would have enhancing effects on emotional resilience.

As hypothesized, interpretation bias as assessed with the recognition task became more positive by CBM-I training compared to CBM-I placebo, although this effect just fell short of significance after Bonferroni-Holm correction. This trend converges with accumulating evidence on the efficacy of CBM-I in changing interpretive style in both adults and adolescents [8], [12], [13], [24]. Note that a neutral control condition was used instead of a negative control condition, and the former has been found to yield smaller or even no effects in previous research (e.g. [46]). Interpretation bias displayed in response to probe scenarios became more positive in both groups, but across sessions the CBM-I group displayed the most positive bias.

Changes in interpretation bias were expected to be accompanied by increased resilience and reduced anxiety and depressive symptoms, but such corresponding effects were not found, neither on the short-term nor on the long-term. Both the CBM-I and Placebo group showed a general decrease in anxiety and depression symptoms over time, but did not differ from each other. Whether this reflects a natural decline, expectancy effects or an unintended positive effect of the placebo training (cf. [23], [47]) is unclear. However, a similar overall decline of anxiety symptoms in adolescents has also been found in a previous study that used a non-intervention instead of a placebo control condition [25]. Thus it seems most parsimonious to attribute the overall decline of symptoms to a natural course. Including the following four moderators: change in interpretation bias, baseline interpretation bias, stressful life events, or number of training-sessions completed, did not change these results. For the secondary emotional outcome measures, the same pattern of general improvement was observed.

In previous CBM-I research, findings on emotional outcomes have also been more mixed [8]. It has been argued that changing interpretive style on a training-related task might be relatively easy, but more time may be necessary for transfer to daily life and emotional symptoms [20]. That is, a change in interpretation bias might only affect emotions in interaction with daily situations. However, the lack of long-term effects in our study (up to one year later), suggests that time alone is not enough to obtain generalization to emotional outcomes. As negative biases have also developed during a long time period and in response to life experiences, more time in between training sessions or including booster sessions might be necessary [48]. The timing of training and assessments is an important issue for further research.

Our study was one of the first to investigate long-term effects of CBM-I in adolescents. The only previous study on CBM-I as a preventive intervention in adolescents similarly found limited effects [24] also at two-year follow up [25], but in a long-term study in adults more promising effects were observed [22]. Although a comparable training paradigm was used, two important differences should be noted here. First, the training in the study of Khalili-Torghabeh et al. [22] was performed in the laboratory rather than online, and CBM effects are generally stronger in such laboratories than online (meta-analysis [7]). This might be related to the lack of experimental control in online studies, which seems especially important in an intervention where task compliance and timing is essential, as is the case with CBM. In contrast, online CBT might suffer less from such distracting environments or technical issues, and small to medium effects on anxiety and depression in young people have been found in a recent meta-analysis [49]. Second, contrary to their pre-selected samples with heightened symptoms, we used unselected adolescents. In our sample, with a relatively low level of symptoms that further decreased over time, it might have been harder to detect any training related changes. We hypothesized that CBM-I would also increase emotional resilience in healthy adolescents, but did not find such effects.

Contrary to our expectations, no correlation was observed between interpretation bias and symptoms of anxiety and depression or secondary emotional measures. This might question the relevance of interpretation bias in unselected samples (compared to (sub-)clinical samples), and thereby undermine the basis of CBM-I as an intervention to increase emotional resilience in such a population. However, the variability in symptom levels in the current sample was high, and previous research has shown that interpretation bias scores on the recognition task are associated with anxiety in adolescents [15]. Therefore, the lack of a correlation in the current study, might be related to limitations of our assessment method. The recognition task was administered in group format, and performance might have suffered from a lack of concentration and motivation. Therefore, the observed scores might not be a fully accurate reflection of existing interpretation biases.

A more general limitation of the recognition task, is its strong resemblance to the CBM-I training task, rendering it vulnerable to practice and demand effects. To investigate transfer effects, it would be necessary to add other tasks that differ more from the training paradigm, like a homophone or face classification task [50]. Previous attempts to demonstrate generalization to other types of interpretation bias assessments have been mainly unsuccessful (e.g. [51], [52]. Note that, although also task-specific, the development of reaction times to probes during training confirmed the change in interpretation bias found on the recognition task.

Another limitation of the current study concerns the high drop-out rates, which are not uncommon in longitudinal and/or online research (cf. [24], [53]). Many participants did not complete the intended amount of training and assessments, and the current results (intention-to-treat approach) might thus not reflect the full potential of multi-session training. Although drop-out was unrelated to emotional functioning at baseline, girls completed relatively more assessment sessions. Therefore, long-term results should be interpreted carefully when referring to adolescent boys. Drop-out at follow-up assessments also reduced power to detect long-term effects, but as we also found no short-term effects, it is unclear whether effects would have been found in a larger sample. Note that we applied a relatively conservative data-analytic approach, including the simultaneous examination of all time points and stringent correction for multiple testing. Although this seems an adequate approach to reduce the risk of Type I errors and to assess robust effects of the intervention, it also reduced power to observe small effects for specific comparisons, particularly in combination with our unbalanced randomization (smaller placebo group).

Although steps were taken to increase engagement and compliance in the current study (by including feedback, progress bar, financial compensation, e-mail, text messages and phone call reminders), more motivating features might be necessary to improve adherence. For example, training might become more appealing when adding gaming elements or a social network environment [54]. This seems especially important in adolescent samples and in healthy samples who may miss an intrinsic motivation to change. Whether intended motivating features indeed increase engagement and adherence, needs to be monitored carefully. Improving adherence is not only relevant for reducing attrition (and increasing representativeness) in intervention studies, but particularly for potential implementation of training paradigms that prove to be effective. Preventive programs should be acceptable for the targeted population, and apart from attractive tasks, this might also require providing a clear rationale [55], which is not current practice in CBM training studies [56]. Whether more explicit instructions (cf. [57]) and psychoeducation will improve efficacy is thus another important question for future research.

To summarize, the CBM-I training was marginally effective in increasing a positive interpretation bias in unselected adolescents, as indexed by both reaction times during probe trials during training and a separate assessment task. However, these changes were not paralleled by a change of any of the emotional measures, neither at the short- nor at the long-term. Consistent with previous findings among adolescents (e.g., [25]), symptoms of anxiety and depression generally decreased over time. Yet, this decline was not especially pronounced in the active condition. Given the limitations of online research (especially in unselected samples), including the high drop-out rates, it would be premature to conclude that CBM-I has no potential, but in its current form, it seems of little use for universal prevention.

## Supporting information

S1 Appendix Additional manipulations and measures. (DOCX)

S1 File CONSORT checklist. (DOCX)

S1 Protocol Trial protocol. (PDF)

S2 Protocol Additional documentation trial protocol. (DOCX)

S3 Protocol Information letters. (PDF)

S4 Protocol Trial protocol translation. (DOCX)
