# Supplementary material for: A randomized controlled trial of multi-session online interpretation bias modification training: Short- and long-term effects on anxiety and depression in unselected adolescents
Source: PLoS One. 2018 Mar 15;13(3):e0194274. doi: 10.1371/journal.pone.0194274 (PMC5854362; doi:10.1371/journal.pone.0194274)
Supplement: S1 Appendix — (DOCX) [file pone.0194274.s001.docx]

**Additional manipulations and measures**

*Training paradigms*

The visual search attentional bias training was based on [1] with some adaptations for adolescents [2]. In this task, participants had to find and select the single happy face in a 4 x 4 grid of negative emotional faces (sad, angry, and fearful; 5 each). To start a trial, participants had to move the mouse cursor over a fixation cross in the center of the screen. The faces were then presented until the participant responded. In case of an erroneous response the trial was repeated after feedback. The task consisted of four blocks with 36 trials each. A progress bar indicated how many trials were left in each block. Between blocks, short breaks were provided with feedback, consisting of the number of points earned based on performance (1-10 points based on RTs for correct trials). Face stimuli were randomly drawn from two sets (counterbalanced over participants) of 36 adolescent faces (18 happy, six fearful, six angry and six sad faces) from the NIMH Child Emotional Faces Picture Set (NIMH_ChEFS, [3], for stimuli selection, see [2]). In the VS placebo condition, participants had to find and select the only 5-petaled flower in a 4 x 4 grid of 7-petaled flowers [1]. Other aspects of the task were identical to the experimental condition.

The original Dot-Probe (DP) attentional bias training [4] was modified for use in child anxiety studies (TAU-NIMH initiative; for details see http://people.socsci.tau.ac.il/mu/anxietytrauma/files/2013/08/TAU-NIMH-ABMT-Project-Overview.pdf). Participants were presented with 160 trials, in which after presentation of a fixation cross in the center of the screen, two faces from the same actor (angry or neutral) were shown on the top and bottom of the screen for 500 ms. Faces from 12 adult actors (six men) from the Nim-Stim stimulus set [5] were used and divided over two sets (counterbalanced over participants). In 120 trials an angry-neutral combination was shown, while the remaining 40 trials were neutral-neutral, to obscure the training contingency. After face presentation, a probe (“<” or “>”) appeared in the location of one of the faces. Participants had to respond with the corresponding arrow key on the keyboard as quickly as possible. The probe was presented until the participant responded. Actor, angry face location and probe direction were fully counterbalanced. In the experimental condition, the probe location was always the location of the neutral face in angry-neutral trials. In neutral-neutral trials, this probe location was random. In the DP placebo condition, actor, angry face location, probe direction and probe location were all fully counterbalanced. Hence, there was no contingency between the location of the probe and the location of the negative and neutral stimuli.

The emotional working memory (EmoWM) training consisted of the chessboard task developed by [6], which was extended with an emotional component for the current study. Participants were presented with a fixation cross, followed by a 4 x 4 matrix of green and blue blocks in chessboard format. Each trial consisted of a sequence of blocks lighting up (900 ms each) and after a light signal participants had to reproduce this sequence by clicking these blocks in the correct order, first clicking all green blocks in the order in which they were presented and then all blue blocks. Blocks lit up in random order, but to ensure that information had to be manipulated in working memory, at least one blue block was presented before the last green block. The emotional component consisted of a negative emotional face which was presented randomly on one of the blocks that lit up and had to be omitted during reproduction. Faces were drawn from the same set as used in the VS training. Each session consisted of 3 training blocks of 12 trials. Sequence length depended on performance, starting with 3 blocks (including the face stimulus) and increasing or decreasing by one block after 2 consecutive correct or incorrect trials respectively. Feedback was provided after each trial, also indicating change in sequence length. In the first training session, 4 practice trials were presented, starting with 2 trials without facial stimuli. In the EmoWM placebo training, the task was non-adaptive and 3 blocks were presented in all trials. Since trials were shorter in duration due to short sequences, each block consisted of 15 trials.

*Questionnaires*

The Attentional Control Scale (ACS, [7]) was used to assess (subjective) attentional control. This 20-item (1-4 scale) self-report questionnaire assesses both attentional focusing and attentional shifting. Cronbach’s alpha *α* = .86 in the current sample.

The Alcohol Use Disorders Identification Test (AUDIT, [8]) was used to assess alcohol use and alcohol-related problems. The 10 self-report items are rated on a 5-point scale (0-4).

The Highly Sensitive Child Questionnaire (HSC Scale, [9]) was used to assess high sensitive personality characteristics. The shortened 12-item version was used, assessing sensitivity to sensory and emotional stimuli and sensitivity to change on a 7-point scale (1-7).

For part of the participants, we assessed responses to real-life stress: that is, they were asked about anxiety and self-efficacy in response to an upcoming exam week. Given the low level of responders and the huge variability in timing and type of exam weeks, we did not analyse these data.

*Assessment of cognitive processes*

Attentional bias was assessed with the Emotional Visual Search Task (EVST) was developed by [2]. The assessment task largely resembled the visual search training, but consisted of two blocks of 36 trials, where participants had to repeatedly select either the only happy face in a 4 x 4 grid of negative faces or the only face with a negative emotion (angry, fearful or sad) in a grid of happy faces. The order of positive or negative blocks was counterbalanced over participants. An attentional bias index was computed by subtracting the average RT for selecting negative faces from the average RT for selecting positive faces.

Attentional bias was also assessed with the Dot-Probe task (DPT), which was almost identical to the dot-probe placebo training (for details on stimuli size, positioning etc., see [10]). However, 120 instead of 160 trials were presented, with 80 neutral-angry trials and 40 neutral-neutral. Actor, angry face location, probe direction and probe location were all fully counterbalanced. An attentional bias index was computed by subtracting average RT for congruent angry-neutral trials (probe at angry location) from incongruent angry-neutral trials (probe at neutral location).

WM capacity was assessed with the Self Ordered Pointing Task (SOPT, [11]). A number of concrete or abstract pictures (increasing from 4 to 12) was presented and participants had to click on each picture only once. However, each time a participants selected a picture, the pictures were reordered. Participants had to remember which pictures they already selected and were not allowed to select the same location twice in a row. The total number of correct clicks was used as an index of WM capacity.

*Saliva collection*

As part of an international collaborative project (TAU-NIMH-ABMT project, http://people.socsci.tau.ac.il/mu/anxietytrauma/files/2013/08/TAU-NIMH-ABMT-Project-Overview.pdf ) we also collected saliva samples for genetic analyses, with the aim of identifying genetic markers for cognitive training efficacy. Adolescents and their parent provided informed consent for the collection of saliva (at T1) in addition to their informed consent for participating in the training study.

**References**

[1] Dandeneau SD, Baldwin MW, Baccus JR, Sakellaropoulo M, Pruessner JC. (2007). Cutting stress of the pass: Reducing vigilance and responsiveness to social threat by manipulating attention. J Pers Soc Psychol. 2007; 93, 651-666. doi: [10.1037/0022-3514.93.4.651](http://psycnet.apa.org/doi/10.1037/0022-3514.93.4.651)

[2] De Voogd EL, Wiers RW, Prins PJM, Salemink E. Visual search attentional bias modification reduced social phobia in adolescents. J Behav Ther Exp Psychiatry. 2014; 45, 252-259. doi: [10.1016/j.jbtep.2013.11.006](http://dx.doi.org/10.1016/j.jbtep.2013.11.006)

[7] Derryberry D, Reed MA. Anxiety-related attentional biases and their regulation by attentional control. J Abnorm Psychol. 2002; 111, 225-236. [doi: 10.1037/0021 -843X.111.2.225](http://ovidsp.tx.ovid.com/sp-3.13.1a/ovidweb.cgi?QS2=434f4e1a73d37e8c61d48fdeff50180f5897c1056073ff384b2ef17fbb22210c706098254fa90444c7853a2ed7f84c9282e2c3ff5aebfa4c62a77400b4e306d21b909b9b06fac7b5ded13bd678babfdab7a4359bae081929f0a05b632bdf7aa104a30a26adec928d32813e045de2f1b3039cf15949f6a60bca209548cada660e153004b9a33bd1c3a7b2dc060ddf962d3433398f102a2f551a54766db91305a00fa886822b70b2783dfc06928131041367fd14a561bda54091ea596eae5d9fb04db5a717c57a2565c7d223fbb1f918f89201761948afa6c830329543eeaed84eefa7768a8d72b03b45e9ffe9e178aa036ecf5770a094ff729a2a522a2ba4292cc30dbd296165da59ff3225c5399164ed186961767b2197ef25818d257df2b64cedc254fc8bb980eb5647d39bc327853bc2565ffe9eafee4f7f16789e737cb0600b56436dc14f626322f721401d55d3d5eff7b0bcaed93f66239f25b9319cd81456dcf1f54ce1f9abc97fbe1ead2583d8fb64980806910197ab0101223614bf4adf59c71c2e81c342)

[6] Dovis S, van der Oord S, Wiers RW, Prins PJM. Can motivation normalize working memory and task persistence in children with Attention-Deficit/Hyperactivity Disorder? The effects of money and computer-gaming. J Abnorm Child Psychol. 2012; 40, 669-681. doi: [10.1007/s10802-011-9601-8](http://link.springer.com/article/10.1007/s10802-011-9601-8)

[3] Egger HL, Pine DS, Nelson E, Leibenluft E, Ernst M, Towbin KE, Angold A. NIMH Child Emotional Faces Picture Set (NIMH-ChEFS): A new set of children’s facial emotion stimuli. Int J Methods Psychiatr Res. 2011; 20, 145–156. doi: [10.1002/mpr.343](http://onlinelibrary.wiley.com/doi/10.1002/mpr.343/full)

[4] MacLeod C, Rutherford E, Campbell L, Ebsworthy G, Holker L. Selective attention and emotional vulnerability: Assessing the causal basis of their association through the experimental manipulation of attentional bias. J Abnorm Psychol. 2002; 111, 107-123. [doi: 10.1037/0021-843X.111.1.107](http://psycnet.apa.org/doi/10.1037/0021-843X.111.1.107%22%20/t%20%22_blank)

[11] Petrides M, Milner B. Deficits on subject-ordered tasks after frontal- and temporal-lobe lesions in man. Neuropsychologia. 1982; 20, 249-262. [doi:10.1016/0028-3932(82)90100-2](http://dx.doi.org/10.1016/0028-3932%2882%2990100-2)

[9] Pluess M, Assary E, Lionetti, F, Lester, K Krapohl, EAron E, Aron A. Environmental sensitivity in children: Development of the Highly Sensitive Child Scale and identification of sensitivity groups. Manuscript in preparation.

[8] Saunders JB, Aasland OG, Babor TF, De La Fuente J, Grant M. Development of the alcohol use disorders identification test (AUDIT): WHO collaborative project on early detection of persons with harmful alcohol consumption-II. Addiction. 1993; 88, 791–803. doi: [10.1111/j.1360-0443.1993.tb02093.x](http://onlinelibrary.wiley.com/doi/10.1111/j.1360-0443.1993.tb02093.x/abstract;jsessionid=1787844B3BE10239DEB474DD23CDC81B.f02t01)

[5] Tottenham N, Tanaka JW, Leon AC, McCarry T, Nurse M, Hare TA, Marcus DJ, Westerlund A, Casey BJ, Nelson C. The NimStim set of facial expressions: Judgments from untrained research participants. Psychiatry Res. 2009; 168, 242-249. doi: [10.1016/j.psychres.2008.05.006](http://dx.doi.org/10.1016/j.psychres.2008.05.006)

[10] **Abend R, Pine DS, Bar-Haim Y. The TAU-NIMH Attention Bias Measurement Toolbox. 2014. Available: http://people.socsci.tau.ac.il/mu/anxietytrauma/research/.**
