# Supplementary material for: A randomized controlled trial of multi-session online interpretation bias modification training: Short- and long-term effects on anxiety and depression in unselected adolescents
Source: PLoS One. 2018 Mar 15;13(3):e0194274. doi: 10.1371/journal.pone.0194274 (PMC5854362; doi:10.1371/journal.pone.0194274)
Supplement: S1 Protocol — (PDF) [file pone.0194274.s003.pdf]

Algemeen deel

Project title

Always look on the bright side of life

Responsible researcher (if a PhD-project is concerned list the professor involved)

Leone de Voogd, Elske Salemink, Reinout Wiers

Who conducts the research? (PhD-students, students, etc.)

Leone de Voogd, Annelot Roorda, Amber Kroon, Daphne Al, Merlijn Koster, Katinka van Zon, Renate Kortekaas

Responsible Department or Section

Developmental Psychology

Research location

Scholen verspreid over Nederland, standplaats Amsterdam

Brief project description (max. 200 words)

Het betreft een onderzoek naar de preventie van angst en depressie en het vergroten van emotionele weerbaarheid bij jongeren van 12-18 jaar, d.m.v. het trainen van cognitieve processen.

Jongeren volgen 2x per week, gedurende 4 weken een online computertraining. Zij worden random toegewezen aan 1 van 5 condities: één van twee aandachtsbiastrainingen, een interpretatietraining, een werkgeheugentraining of een placebotraining.

Voor en na de trainingsperiode worden de cognitieve processen gemeten en vragenlijsten ingevuld over het emotioneel functioneren. Op de voormeting wordt daarnaast speeksel verzameld voor genetisch onderzoek. Op de nameting wordt een sociale stresstaak afgenomen. Deze metingen vinden plaats op school.

Na 3,6, en 12 maanden worden follow-up metingen verricht, bestaande uit vragenlijsten. Ook zal rondom de eerste proefwerkweek na de training een vragenlijst afgenomen worden.

Expected duration of the project

16 maanden

Expected number of participants

600

This project is comparable with the following submitted project(number)

2012-DP-2251

Inhoudelijk deel

A1. When classifying the research as Medical vs. Non-medical, does it comply with A1, meaning it can be listed under category D (see also Appendix 1, 2.4)?

Yes, it falls into category D

A2. Are consenting adults selected, as described in A2?

No, describe participant details and recruitment procedure
Please submit the information letter and the consent form as attachment

Comment:

Het onderzoek wordt uitgevoerd onder jongeren in de leeftijd van 12 tot 18 jaar. Zij worden benaderd via hun school en doen mee na actieve toestemming van henzelf en een ouder/verzorger.

A3. Are participants free to decide to participate and to stop for whatever reason, as listed under A3?

Yes

A4. Are participants subjected to a screening procedure to reduce the risks for adverse effects, as listed under A4?

No

Comment:

Er zijn geen exclusiecriteria bij dit onderzoek en de risicos zijn verwaarloosbaar.

A5. Is there a risk for chance incidents that should be reported to the participant, as listed under A5?

In doubt, because

Comment:

Uit de gebruikte vragenlijsten kan blijken dat een proefpersoon last heeft van angstige of depressieve klachten, al kan geen diagnose gesteld worden.
Wanneer jongeren boven de klinische cut-off score uitkomen, nemen wij contact met hen en hun ouders op.

A6. Are participants fully informed before participating, and do they sign a consent form, as listed under A6?

Yes, please submit the information letter and the consent form as attachment

A7. Is participant privacy and anonymity guaranteed, as listed under A7?

Yes

A8. In case of deception, does the procedure comply with the conditions listed under A8? (full disclosure concerning risks, accurate debriefing)?

The deception complies with the conditions listed under A8
(Please submit a description of the debriefing procedure, e.g., a debriefing document for the participant)

A9. Is there a risk that a substantial number of participants will drop out because the research is considered to be discomforting, as listed under A9?

No

B1. Does the research **fully** comply with the guidelines for Standard Research?

Yes, specify below and upload a concise research description (max 1 A4)

Answer:

B3. Psychofysica / Gedragstaken
