# Supplementary material for: A randomized controlled trial of multi-session online interpretation bias modification training: Short- and long-term effects on anxiety and depression in unselected adolescents
Source: PLoS One. 2018 Mar 15;13(3):e0194274. doi: 10.1371/journal.pone.0194274 (PMC5854362; doi:10.1371/journal.pone.0194274)
Supplement: S2 Protocol — (DOCX) [file pone.0194274.s004.docx]

Additional information A2

For information letters see S3 Protocol

Toestemmingsverklaring

**Deze verklaring dient door zowel de leerling als de ouder/verzorger ingevuld en ondertekend te worden!**

**Persoonlijke gegevens leerling**

| Naam: M / V |
| --- |
| Geboortedatum: |
| Telefoon: |
| E-mail adres: |
| Telefoon ouder/verzorger: |
| E-mail adres ouder/verzorger: |

**Toestemming door leerling**

Ja, ik doe mee aan het onderzoek ‘Always look on the bright side of life’ van de Universiteit van Amsterdam. Ik heb de informatie over het onderzoek gelezen en begrepen en weet dat ik mijn toestemming kan intrekken en mijn deelname kan stoppen wanneer ik maar wil.

Ik wil ook speeksel afgeven voor genetisch onderzoek: Ja / Nee

Datum: Handtekening leerling:

Plaats:

--------------------------------------------------------------------------------------------------------------------------------------**Toestemming door ouder/verzorger**

Ja, ik geef mijn zoon/dochter toestemming om mee te doen aan het onderzoek ‘Always look on the bright side of life’ van de Universiteit van Amsterdam. Ik heb de informatie over het onderzoek gelezen en begrepen. Ik heb te allen tijde het recht om mijn toestemming in te trekken en de deelname van mijn zoon/dochter aan het onderzoek te laten stoppen.

Ik verleen ook toestemming voor speekselafname ten behoeve van genetisch onderzoek: Ja / Nee

Naam ouder/verzorger:

Datum: Handtekening ouder/verzorger:

Plaats:

Additional information A8

Omschrijving debriefing

De stresstaak (Cyberball, zie toelichting details) wordt uitgevoerd als laatste onderdeel van de nameting (gevolgd door een mood scale). Zodra alle proefpersonen de nameting hebben afgerond, worden zij ingelicht over de inhoud van de stresstaak.

Hen wordt verteld dat zij niet daadwerkelijk met andere jongeren speelden, maar dat het spel bepaald werd door de computer. Ook wordt verteld dat iedereen hierbij buitengesloten werd tijdens het spel en dat de proefpersoon hier dus geen invloed op had.

Daarbij wordt ruimte gelaten voor vragen of opmerkingen en worden leerlingen uitgenodigd de onderzoeker na afloop aan de spreken, mochten zij toch nog last hebben van de taak.

De sessie wordt afgesloten met het uitdelen van de eerste beloningen, voor de meeste deelnemers een positieve ervaring.

Additional information B1

Procedure

Leerlingen uit de geselecteerde klassen krijgen in de klas een informatiefolder mee. Hun ouders krijgen via school een brief of e-mail met informatie en een toestemmingsformulier. Zij kunnen dit formulier nadat zij het met hun kind ondertekend hebben op school inleveren of naar de UvA sturen. Nadat het formulier is ontvangen, krijgen de leerlingen een e-mail om zich online te registreren voor het onderzoek. Het onderzoek start met een voormeting op school van 90 minuten. Enkele dagen later zal de eerste training van 15 minuten op school plaats vinden. De overige 7 trainingen (2 per week) worden thuis of op school via het internet gevolgd. Na de 4 weken vindt een nameting plaats op school van wederom 90 minuten.

Voor en tijdens de eerstvolgende proefwerkweek, krijgen leerlingen en vragenlijst gemaild.

Na 3,6, en 12 maanden wordt hen nogmaals gevraagd online vragenlijsten in te vullen.

Leerlingen worden beloond d.m.v. cadeaubonnen en deelname aan een loterij. Afhankelijk van hun deelname aan een deel van of alle sessies, kan dit oplopen tot 17,50 euro aan cadeaubonnen en kans op een prijs zoals een I-pod.

Materialen voor- en nameting:

Assessmentversies van de aandachtbiastrainingstaken en interpretatietrainingstaak.

Self Ordered Pointing Task (werkgeheugentaak met neutrale afbeeldingen)

Vragenlijsten: angst/depressie, zelfvertrouwen, piekeren, algemene emotionele en gedragsproblemen, middelengebruik, aandachtscontrole, ervaren stress, faalangst, weerbaarheid.

Stresstaak (alleen nameting): Cyberball, waarbij de proefpersoon een virtueel balspel speelt met zogenaamd 2 andere leerlingen. Deze spelers worden gestuurd door de computer en de proefpersoon wordt daarbij buitengesloten.

Speekselafname (alleen voormeting): proefpersonen vullen buiten het klaslokaal (in 2- of 3-tallen) één buisje met speeksel. Dit wordt gecodeerd verzonden naar King’s College London, waar het materiaal opgeslagen wordt en de genetische analyses plaatsvinden. Voor de speekselafname wordt apart toestemming gevraagd aan leerlingen en ouders.

Trainingstaken

Leerlingen worden random toegewezen aan één van vier trainingsgroepen:

1. Aandachtbiastraining

*Deelnemers zoeken een positief gezicht in een 4x4 matrix van negatieve gezichten.*

1. Aandachtbiastraining

*Dot probe taak: deelnemers reageren op een probe die achter een neutraal gezicht verschijnt.*

1. Interpretatietraining

*Deelnemers lezen ambigue scenario’s, waarbij zij positieve woordfragmenten aan moeten vullen.*

1. Chessboard training (werkgeheugen)

*Deelnemers onthouden en reproduceren de locatie en volgorde van (een toenemend aantal) oplichtende blokjes in een 4x4 schaakbord. Er worden afleidende emotionele gezichten getoond.*

1. Placebotraining (placeboversie van één van de 4 trainingen)

Deelnemers weten dat van sommige trainingen meer effect wordt verwacht dan andere, maar weten niet welke training zij krijgen.

Stimuli

Bij de verschillende trainingen en metingen van aandachtbias wordt gebruik gemaakt van de volgende emotionele stimuli:

- Blije, bange, boze en verdrietige gezichten uit de NIMH Child Emotional Faces Picture Set.

- Neutrale en boze gezichten uit de NimStim Set en twee gezichten uit de Matsumoto & Ekman set.
