# Supplementary material for: A randomized controlled trial of multi-session online interpretation bias modification training: Short- and long-term effects on anxiety and depression in unselected adolescents
Source: PLoS One. 2018 Mar 15;13(3):e0194274. doi: 10.1371/journal.pone.0194274 (PMC5854362; doi:10.1371/journal.pone.0194274)
Supplement: S3 Protocol — (PDF) [file pone.0194274.s005.pdf]

# *Always look on the bright side of life*

Preventieonderzoek Universiteit van Amsterdam

## Geachte ouder/verzorger,

Het Baken Stad College heeft aangegeven mee te willen werken aan een onderzoek van de Universiteit van Amsterdam (UvA) naar een preventieprogramma ter vergroting van de emotionele weerbaarheid bij jongeren: “Always look on the bright side of life”. Graag willen wij uw toestemming vragen voor deelname van uw zoon/dochter aan dit onderzoek.

## Waarom doen we dit onderzoek?

Een groot deel van de middelbare scholieren voelt zich wel eens angstig of somber. Bij zo’n 10-20% van de jongeren zijn deze gevoelens zo sterk dat zij een angststoornis of depressie ontwikkelen. Deze klachten beperken de kwaliteit van leven in sterke mate. Daarnaast gaan deze problemen bij jongeren vaak samen met slechtere schoolprestaties en andere problemen, zoals middelen-gebruik of gedragsproblemen. Het is dan ook van groot belang om tijdig in te grijpen bij klachten of deze zelfs voor te zijn.

## Waar gaat het onderzoek over?

Het onderzoek, uitgevoerd door de afdeling ontwikkelingspsychologie van de UvA, richt zich op het versterken van emotionele weerbaarheid. Ons doel is de kans op het ontstaan van angst en depressie te verkleinen én gezonde jongeren weerbaarder te maken tegen stress. Jongeren die kwetsbaar zijn voor negatieve gevoelens hebben de neiging hun aandacht selectief te richten op negatieve informatie en hebben moeite om hun negatieve gedachten onder controle te krijgen. Het onderzoek richt zich op het veranderen van deze patronen van informatieverwerking. Door middel van een computertraining leren jongeren om positiever naar hun omgeving te kijken en meer controle uit te oefenen op hun denken.

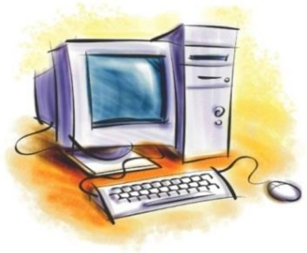

## Wat houdt het onderzoek in?

Gedurende vier weken volgen de jongeren 2 x per week een training van ongeveer 15 minuten. De eerste training volgen zij op school en de overige trainingen kunnen zij thuis via het internet volgen. Hier worden zij aan herinnerd door middel van e-mail en SMS.

Om het effect van de trainingen te meten, vullen de jongeren vragenlijsten in over hun gevoelens en manier van denken en worden diverse computertaken afgenomen. Deze metingen vinden vóór en na de trainingsperiode plaats op school en zullen beide keren ongeveer 90 minuten duren.

3, 6 en 12 maanden na afronding van de training zal uw zoon/dochter opnieuw gevraagd worden enkele vragenlijsten in te vullen. De eerste van deze metingen zal op school plaatsvinden en de overigen via het internet. Tot slot zal rond een proefwerkweek of examen nog één vragenlijst via internet ingevuld worden.

Behalve van uw zoon/dochter, ontvangen we ook van u graag enkele gegevens. U zult op de voormeting gevraagd worden naar enkele demografische gegevens. Daarnaast vragen wij u op de voormeting, nameting en follow-up metingen één vragenlijst in te vullen over het gedrag van uw zoon/dochter. Hierdoor krijgen wij een breder beeld van de emotionele weerbaarheid van de deelnemende jongeren.

Universiteit van Amsterdam

**De planning op de school van uw zoon/dochter ziet er o.v.b. van roosterwijzigingen als volgt uit:**

| | |
|---|---|
| **Eind februari/begin maart** | **Voormeting (90 min)** |
| **De hele maand maart** | **8 Trainingen (10-20 min)** |
| **Eind maart/begin april** | **Nameting (90 min)** |
| **Begin juli 2013** | **Follow-up 1 (30 min)** |
| **Begin oktober 2013** | **Follow-up 2 (30 min)** |
| **April 2014** | **Follow-up 3 (30 min)** |

Tot slot wordt uw zoon/dochter ook gevraagd deel te nemen aan een genetisch onderzoek. Hierover leest u meer onder het kopje “deelname aan genetisch onderzoek”. Voor dit onderdeel kunt u apart besluiten om toestemming te geven.

Het onderzoek ’Always look on the bright side of life’ is goedgekeurd door de Commissie Ethiek van de Afdeling Psychologie van de Universiteit van Amsterdam. Er zijn geen risico’s verbonden aan deelname aan dit onderzoek.

## Deelname genetisch onderzoek

Als onderdeel van dit onderzoek wordt uw zoon/dochter ook gevraagd om deel te nemen aan een genetisch onderzoek. Het doel van dit onderzoek is te kijken of bepaalde genen voorspellend kunnen zijn voor emotionele klachten en voor het succes van onze trainingen. Dit onderzoek wordt uitgevoerd in samenwerking met Dr. Thalia Eley, Institute of Psychiatry, London, UK; Prof. Yair-Bar-Haim, Tel Aviv University, Israel en Prof. Daniel Pine, National Institute for Mental Health, Washington, USA.

We zullen uw zoon/dochter vragen een kleine hoeveelheid speeksel af te geven tijdens de voormeting op school. Een onderzoeksassistent zal hem/haar hierbij begeleiden en precies vertellen wat de bedoeling is. Uw kind zal in een klein plastic buisje spugen. Dit duurt slechts enkele minuten.

Uit dit speeksel kan het DNA afgeleid worden. We zullen bepaalde genen analyseren die eerder in verband gebracht zijn met emotionele klachten en behandeleffecten.

Het speeksel van uw kind zal na afloop van het project vernietigd worden. De DNA-gegevens worden anoniem opgeslagen en bewaard voor eventueel toekomstig door de Commissie Ethiek goedgekeurd onderzoek. De identiteit van uw kind wordt beschermd in alle publicaties. De gegevens worden opgeslagen onder een code, zodat we ze wel kunnen koppelen aan de andere gegevens die we van uw kind verzamelen.

U ontvangt geen verslag van de analyse van het DNA van uw kind. Wel ontvangt u een verslag van de uitkomsten van het onderzoeksproject. Op basis van dit onderzoek kunnen geen uitspraken gedaan worden over de medische toestand of gezondheidsrisico’s van uw kind. Deelnemers delen niet in de rechten van ontdekkingen op basis van dit onderzoek.

Indien u toestemming geeft voor het genetische onderdeel van dit onderzoek, gaat u akkoord met het gebruik en het opslaan van de geanonimiseerde DNA-gegevens van uw kind voor het huidige onderzoek en voor toekomstig door de Commissie Ethiek goedgekeurd onderzoek.

## Wat gebeurt er met de gegevens van uw zoon/dochter?

Alle gegevens worden anoniem en strikt vertrouwelijk behandeld. Deelname is natuurlijk geheel vrijwillig en u of uw zoon/dochter kunnen op ieder gewenst moment de medewerking opzeggen. Onze ervaring is dat leerlingen het leuk vinden om mee te doen . De onderzoeksresultaten worden gebruikt om uitspraken te kunnen doen over groepen en er zal dan ook niet gekeken worden naar de individuele resultaten van uw zoon/dochter. Alleen wanneer uw zoon/dochter op de vragenlijsten aangeeft vaak negatieve emoties te ervaren, zullen wij persoonlijk contact met u en uw kind opnemen.

## Hoe kunt u aangeven dat uw zoon/dochter mee mag doen?

Bij deze brief zit een toestemmingsformulier. Zowel u als uw zoon/dochter moeten toestemming geven voor deelname aan het onderzoek. U kunt hierop ook aangeven of u wel of niet toestemming geeft voor het genetische onderdeel van het onderzoek. Dit heeft geen consequenties voor deelname aan de rest van het onderzoek. Wanneer u beiden getekend heeft, kunt u het formulier inleveren op school of inscannen/fotograferen en mailen naar E.L.deVoogd@uva.nl

## Hoe kunt u aangeven dat uw zoon/dochter niet mee mag/wil doen?

Indien u of uw zoon/dochter niet mee wil doen aan het onderzoek, hoeft u het formulier niet terug te sturen. Tijdens het onderzoek zal uw zoon/dochter dan een andere opdracht krijgen van de begeleidende docent. Ook als u of uw kind gedurende het onderzoek niet meer mee wilt doen, kan dit ten alle tijde worden aangegeven bij de aanwezige onderzoekers.

## Wat heeft u/uw kind aan het onderzoek?

Met uw deelname draagt u bij aan het ontwikkelen van een effectief preventie-programma waarvan jongeren en hun ouders/begeleiders uiteindelijk kunnen profiteren. Daarnaast heeft het volgen van de trainingen mogelijk een positief effect op de emotionele weerbaarheid van uw zoon/dochter. Tot slot ontvangen de jongeren cadeaubonnen (tot max. 17,50 voor het gehele onderzoek) voor deelname aan het onderzoek en doen zij mee aan een loterij, waarbij zij kans maken op een mooie prijs.

## Heeft u nog vragen?

Voor vragen over het onderzoek kunt u contact opnemen met Leone de Voogd, tel: 020-5256909 / e-mail: E.L.deVoogd@uva.nl. Voor klachten of vragen of opmerkingen die u niet direct met de onderzoeker wilt bespreken, kunt u contact opnemen met dr. Van den Wildenberg, lid van de Commissie Ethiek, tel: 020-5256686 / e-mail: W.P.M.vandenWildenberg@uva.nl.

**Wij hopen dat u en uw zoon/dochter mee willen werken aan dit onderzoek. Hiermee levert u een belangrijke bijdrage aan het verhogen van de emotionele weerbaarheid van jongeren.**

Met vriendelijke groet,

Leone de Voogd, MSc. (hoofdonderzoeker)
Dr. Elske Salemink

Universiteit van Amsterdam, Programmagroep Ontwikkelingspsychologie
Adres: Weesperplein 4, 1018 XA Amsterdam
Telefoon: 020-5256909
Email: E.L.deVoogd@uva.nl

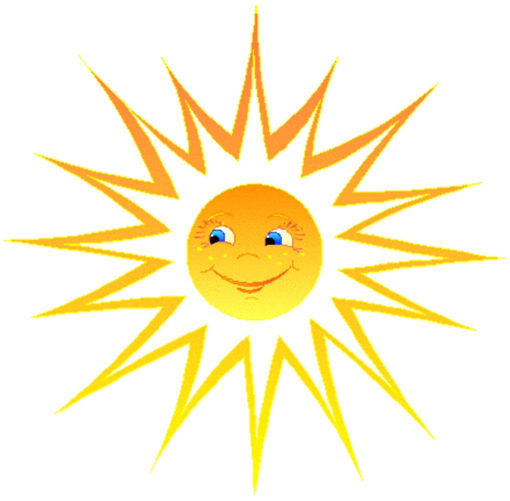

NB: Deze brief heeft u via de school van uw zoon/dochter ontvangen. Wij zijn niet in het bezit van persoonlijke gegevens van u of uw kind. Deze ontvangen wij pas van u, wanneer u toestemming geeft via het toestemmingsformulier.

Dit onderzoek wordt mede mogelijk gemaakt door:
ZonMw

# *Always look on the bright side of life*

Preventieonderzoek Universiteit van Amsterdam

## Beste leerling,

Jouw school werkt mee aan een onderzoek van de afdeling ontwikkelingspsychologie van de Universiteit van Amsterdam, "Always look on the bright side of life". Het onderzoek gaat over een preventietraining waarmee we jongeren willen leren beter met stress en lastige situaties om te gaan. Met deze brief willen we jou vragen of je ook mee wilt doen aan dit onderzoek.

## Waarom dit onderzoek?

Veel jongeren voelen zich wel eens zenuwachtig of een beetje somber. Bijvoorbeeld als ze een spreekbeurt moeten houden, of als ze ruzie hebben met een vriend(in). Sommige jongeren hebben zoveel last van deze gevoelens dat ze het moeilijk vinden om hun schoolwerk te maken of om plezier te hebben. Daarom is het belangrijk om op tijd iets te doen aan dit soort gevoelens.

## Wat houdt het onderzoek in?

Wij doen onderzoek naar een computertraining, waarin jongeren leren met een positieve bril naar hun omgeving te kijken en meer controle te krijgen over hun gedachten en gevoelens. Wij verwachten dat dit soort trainingen helpen om beter met stress en negatieve gevoelens om te gaan.

Als je meedoet aan dit onderzoek, zal je 4 weken lang 2x per week een computertraining volgen van ongeveer 15 minuten. De eerste training volg je op school, de rest doe je thuis via het internet op een moment dat het jou uitkomt. We zullen je hieraan herinneren per e-mail en SMS.

Vóór en na de trainingsperiode vul je op school verschillende vragenlijsten in over je gevoelens en gedachten. Ook doe je dan verschillende computertaakjes. Dit duurt beide keren ongeveer 90 minuten.

3,6, en 12 maanden na afloop van de training vragen we je nogmaals via internet een aantal vragenlijsten in te vullen. Ook zal je rondom het examen nog een keer een e-mail krijgen om een vragenlijst in te vullen. We zullen ook je ouders/verzorgers vragen een paar keer een vragenlijst in te vullen.

Tot slot vragen we je ook deel te nemen aan een genetisch onderzoek. Hierover lees je meer onder het kopje "deelname aan genetisch onderzoek". Voor dit onderdeel kun je apart besluiten om toestemming te geven.

**De planning op jouw school ziet er o.v.b. van roosterwijzigingen als volgt uit:**

| | |
|---|---|
| **Voormeting (90 min)** | **eind februari/begin maart** |
| **Trainingen (10-20 min)** | **de hele maand maart** |
| **Nameting (90 min)** | **eind maart/begin april** |
| **Follow-up 1 (30 min)** | **begin juli** |
| **Follow-up 2 (30 min)** | **begin oktober** |
| **Follow-up 3 (30 min)** | **april 2014** |

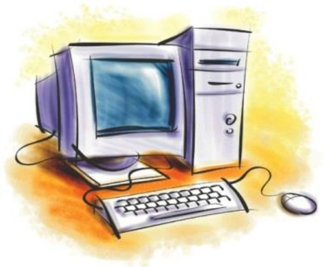

Universiteit van Amsterdam

## Deelname genetisch onderzoek

Als onderdeel van dit onderzoek vragen we je om deel te nemen aan een genetisch onderzoek. Hiermee willen we kijken of bepaalde genen voorspellen of je emotionele klachten krijgt en of je iets hebt aan de trainingen.

We vragen je hiervoor een kleine hoeveelheid speeksel af te geven tijdens de voormeting op school. Een onderzoeksassistent zal je hierbij begeleiden en precies vertellen wat de bedoeling is. Je zal in een klein plastic buisje spugen. Dit duurt slechts enkele minuten.

Met dit speeksel kan gekeken worden wat je DNA is. Je speeksel wordt na afloop van het onderzoek vernietigd en de informatie over jouw DNA wordt anoniem bewaard.

## Wat gebeurt er met mijn gegevens?

De gegevens die jij invult, krijgt niemand anders dan de onderzoekers te zien. Wij kijken alleen naar de resultaten van verschillende groepen jongeren. Alleen wanneer je op de vragenlijsten aangeeft dat je veel last heb van negatieve gevoelens, zullen wij persoonlijk contact opnemen met jou en je ouders.

## Wat heb ik eraan?

Als je meedoet aan het onderzoek, leer je misschien wel beter met stress en negatieve gevoelens om te gaan. Dat kan bijvoorbeeld helpen bij het maken van spannende toetsen of bij andere voor jou stressvolle situaties. Ook help je ons een goede preventietraining te ontwikkelen, waar andere jongeren later weer iets aan hebben.

Om je hiervoor te bedanken, krijg je cadeaubonnen voor je deelname aan het onderzoek. Als je álle trainingen en metingen meedoet, kun je 17,50 euro verdienen. Daarnaast doe je mee aan een loterij waarin je leuke prijzen kunt winnen.

## Wat moet ik nu doen?

Als je mee wilt doen aan het onderzoek, moet je samen met je ouders/verzorgers het toestemmingsformulier invullen en inleveren op school. Je kunt hierop ook invullen of je wel of niet speeksel af wilt geven voor het genetische onderzoek. Als wij het formulier hebben ontvangen, mailen we je een link om je online te registreren voor het onderzoek.

Als je niet mee wilt doen, hoef je helemaal niets te doen. Je docent zal je dan vertellen wat je kunt doen terwijl jouw klas meedoet aan het onderzoek. Als je tijdens het onderzoek liever niet meer mee wilt doen, kan dit altijd. Je kunt dit dan tegen de aanwezige onderzoeker zeggen.

**Ik heb nog een vraag…**

Als je nog vragen hebt, kijk dan eerst met je ouders in de brief die zij gekregen hebben. Als je dan nog meer wilt weten, kunnen jullie contact opnemen met Leone de Voogd, tel: 020-5256909 / email: E.L.deVoogd@uva.nl. Heb je klachten over het onderzoek of vragen die je niet met de onderzoeker zelf wilt bespreken? Neem dan contact op met dr. Van den Wildenberg, lid van de Commissie Ethiek, tel: 020-5256686 / email: W.P.M.vandenWildenberg@uva.nl.

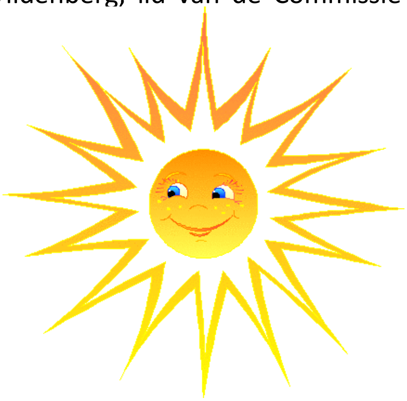

We hopen dat we van je horen!

Met vriendelijke groet,

Leone de Voogd, MSc. (hoofdonderzoeker)
Dr. Elske Salemink

Universiteit van Amsterdam, Programmagroep Ontwikkelingspsychologie
Adres: Weesperplein 4, 1018 XA Amsterdam
Telefoon: 020-5256909
Email: E.L.deVoogd@uva.nl

Dit onderzoek wordt mede mogelijk gemaakt door:
ZonMw
