# Supplementary material for: A randomized controlled trial of multi-session online interpretation bias modification training: Short- and long-term effects on anxiety and depression in unselected adolescents
Source: PLoS One. 2018 Mar 15;13(3):e0194274. doi: 10.1371/journal.pone.0194274 (PMC5854362; doi:10.1371/journal.pone.0194274)
Supplement: S4 Protocol — (DOCX) [file pone.0194274.s006.docx]

**Ethical Committee Proposal 2012-DP-2492**

*Project title*

*Always look on the bright side of life*

*Responsible researcher (if a PhD-project is concerned list the professor involved)*

*Leone de Voogd, Elske Salemink, Reinout Wiers*

*Who conducts the research? (PhD-students, students, etc.)*

*Leone de Voogd, Annelot Roorda, Amber Kroon, Daphne Al, Merlijn Koster, Katinka van Zon, Renate Kortekaas*

*Responsible Department or Section*

*Developmental Psychology*

*Research location*

*Schools in the Netherlands, main research location Amsterdam*

*Brief project description (max. 200 words)*

*The project concerns a study on preventing anxiety and depression and increasing emotional resilience in adolescents aged 12-18 years by training cognitive processes.*

*Adolescents will receive online computer training twice a week, during 4 weeks.*

*They will be randomly allocated to one of 5 conditions: one of two attentional bias trainings, an interpretation bias training, a working memory training, or a placebo training.*

*Before and after the training period, cognitive processes will be assessed and questionnaires on emotional functioning will be completed. At the pre-training assessment, saliva samples will be collected for genetic research and at the post-training assessment a stress task will be performed. These assessments will be completed at school.*

*After 3, 6 and 12 months, follow-up assessments will be completed, consisting of questionnaires. Also, around the first exam week after training, a short questionnaires will be administered.*

*Expected duration of the project*

*16 months*

*Expected number of participants*

*600*

*This project is comparable with the following submitted project(number)*

*2012-DP-2251*

*A1. When classifying the research as Medical vs. Non-medical, does it comply with A1, meaning it can be listed under*

*category D (see also Appendix 1, 2.4)?*

*Yes, it falls into category D*

*A2. Are consenting adults selected, as described in A2?*

*No, describe participant details and recruitment procedure. Please submit the information letter and the consent form as attachment*

Note to readers: information letters are uploaded in Dutch (Supplement 3c)

Informed consent

**This consent form should be filled out and signed by both the student and the parent/caregiver!**

**Personal information student**

| Name: M / F |
| --- |
| Date of birth: |
| Phone: |
| E-mail address: |
| Phone parent/caregiver: |
| E-mail address parent/caregiver: |

**Informed consent student**

Yes, I agree to participate in the study ‘Always look on the bright side of life’ from the University of Amsterdam. I have read and understood the information about the study and I understand that I can cancel this agreement and stop participating at any time.

I also want to donate saliva for genetic research: Yes / No

Date: Signature student:

Place:

--------------------------------------------------------------------------------------------------------------------------------------

**Informed consent parent/caregiver**

Yes, I give my son/daughter permission to participate in the study ‘Always look on the bright side of life’ from the University of Amsterdam. I have read and understood the information about the study. I have the right to cancel this agreement and to let my son/daughter stop participating at any time.

I also provide consent for saliva collection for genetic research: Yes / No

Name parent/caregiver:

Date: Signature parent/caregiver:

Place:

*Comment:*

*The study will be conducted with adolescents aged 12 to 18 years. They will be recruited via schools and only participate after active consent from the adolescent and one parent/ caregiver.*

*A3. Are participants free to decide to participate and to stop for whatever reason, as listed under A3?*

*Yes*

*A4. Are participants subjected to a screening procedure to reduce the risks for adverse effects, as listed under A4?*

*No*

*Comment:*

*There are no exclusion criteria in this study and associated risks are negligible.*

*A5. Is there a risk for chance incidents that should be reported to the participant, as listed under A5?*

*In doubt, because*

*Comment:*

*The questionnaires used can indicate that a participant suffers from anxiety or depressive symptoms, although no diagnosis can be given.*

*If adolescents score above the clinical cut-off, we will contact these adolescents and their parents.*

*A6. Are participants fully informed before participating, and do they sign a consent form, as listed under A6?*

*Yes, please submit the information letter and the consent form as attachment*

*A7. Is participant privacy and anonymity guaranteed, as listed under A7?*

*Yes*

*A8. In case of deception, does the procedure comply with the conditions listed under A8? (full disclosure concerning risks,accurate debriefing)?*

*The deception complies with the conditions listed under A8*

*The stress task (Cyberball, see additional details) will be performed as the last task of the post-training assessment (followed by mood scales). As soon as all participants in a room have completed the assessment, they will be informed about the content of the stress task. They will be told that they did not really play with other adolescents, but that the computer determined the game. They will also be told that all participants were excluded during the game and that they did not have any influence on this. There will be room for questions or comments and participants will be encouraged to talk to the research assistant afterwards if they feel bad about the task. The assessment session will be closed by distributing the first rewards, which will be a positive experience.*

*A9. Is there a risk that a substantial number of participants will drop out because the research is considered to be discomforting, as listed under A9?*

*No*

*B1. Does the research* **fully** *comply with the guidelines for Standard Research?*

*Yes, specify below and upload a concise research description (max 1 A4):*

*Answer:*

*B3. Psychophysiological measures / Behavioral tasks*

***Research description***

*Procedure*

*Students from selected school classes receive an information letter in class, and their parents receive another information letter and informed consent form by e-mail or post. After signing the form, they can either hand it in at school or sent it to the University of Amsterdam. Once the informed consent is received, students receive an e-mail to create an online account. The study starts with a pre-training assessment at school of 90 minutes. Several days later, the first training session of 15 minutes will be performed at school. The other 7 sessions (twice a week) will be performed online at home or at school. After 4 weeks, the post-training assessment will take place at school, again taking 90 minutes. Before and after the first coming exam week, participants will be asked to complete some other online questionnaires. Participants will be rewarded with vouchers and participation in a lottery. Depending on their participation in some or all sessions, this may reach a total amount of 17,50 euro and the chance to win an I-pod.*

*Assessment materials*

*Assessment versions of the attentional bias training tasks and the interpretation training task.*

*Self Ordered Pointing Task (Working memory task with neutral pictures)*

*Questionnaires: Anxiety/depression, self-esteem, worry, general emotional and behavioral problems, substance use, attentional control, stress, test anxiety, resilience.*

*Stress task: Cyberball, in which participants play a virtual ball tossing game and are led to believe that they play with 2 other students. They actually play with the computer and the game is programmed such that the participant is excluded from the game.*

*Saliva collection: Participants will fill a tube with saliva, outside of the classroom (with 2 or 3 students at a time). Saliva samples will be sent anonymously (coded) to King’s College London for storage and analysis. Adolescents and parents will be asked to provide informed consent separately for saliva collection.*

*Training tasks*

*Participants will be randomly allocated to one of four training groups:*

1. Attentional bias training

*Participants search for one positive face in a 4x4 matric of negative faces*

1. Attentional bias training

*Dot-probe task: participants respond to a probe that replaces a neutral face*

1. Interpretation training

*Participants read ambiguous scenarios and complete positive word-fragments*

1. Chessboard training (working memory)

*Participants remember and reproduce the location and order of (an increasing number of) squares that lit up on a 4x4 chessboard. Distracting emotional faces will be presented.*

1. Placebo training (placebo variant of one of the 4 training tasks)

*Participants know that more effects are expected from some training tasks than from others, but do not know which kind of training they receive.*

*Stimuli*

*For the training and assessment of attentional bias, the following stimuli will be used:*

- *Happy, fearful, angry and sad faces from the NIMH Child Emotional Faces Picture Set.*
- *Neutral and angry faces form the NimStim Set and two faces from the Masumoto & Ekman set.*
